# Digital health tools applications in frail older adults—a review article

**DOI:** 10.3389/fdgth.2025.1495135

**Published:** 2025-03-03

**Authors:** Natthanaphop Isaradech, Wachiranun Sirikul

**Affiliations:** ^1^ Department of Community Medicine, Faculty of Medicine, Chiang Mai University, Chiang Mai, Thailand; ^2^ Center of Data Analytics and Knowledge Synthesis for Health Care, Chiang Mai University, Chiang Mai, Thailand; ^3^ Environmental and Occupational Medicine Excellence Center, Faculty of Medicine, Chiang Mai University, Chiang Mai, Thailand

**Keywords:** digital health tools, frailty, geriatrics, prevention, older adults

## Abstract

**Introduction:**

Frailty is a common degenerative condition highly prevalent in adults over 65 years old. A frail person has a higher risk of morbidities and mortality when exposed to health-related stressors. However, frailty is a reversible state when it is early diagnosed. Studies have shown that frail people who participated in an exercise prescription have a greater chance to transition from frail to fit. Additionally, with a rapid advancement of technology, a vast majority of studies are supporting evidence regarding the digital health tools application on frail population in recent years.

**Methods:**

This review comprehensively summarizes and discusses about technology application in frail persons to capture the current knowledge gaps and propose future research directions to support additional research in this field. We used PubMed to search literature (2012–2023) with pre-specified terms. Studies required older adults using digital tools for frailty comparison, association, or prediction and we excluded non-English studies and those lacking frailty comparison or digital tool use.

**Results:**

Our review found potential etiognostic factors in trunk, gait, upper-extremity, and physical activity parameters for diagnosing frailty using digital tools in older adults.

**Conclusion:**

Studies suggest exercise improves frailty status, emphasizing the need for integrated therapeutic platforms and personalized prevention recommendations.

## Introduction

Aging society is an inevitable ongoing trend in the world. The definition, pace, and implications of aging differ significantly between developing and developed countries. In developing countries, the elderly are typically defined as those aged 60 and above, compared to 65 in developed regions, due to variations in life expectancy, socioeconomic conditions, and healthcare access (1–6). In developing regions, the elderly population is growing 1.5 times faster and faces greater challenges, including poverty, poor health, and limited social support systems (7–9). In contrast, developed countries benefit from established healthcare, welfare systems, and healthier, more active elderly populations (10). World Health Organization reported that within 2030 1 in 6 people in the world will be aged over 65 years and by 2050 number of persons aged over 60 years old is expected to reach 426 million (6, 11). This trend is common in many countries and is attributed to a combination of factors such as improved healthcare and advancements in medical technology, which have allowed people to live longer (12). The world public health is now facing the challenges and opportunities that come with an aging society since older adults are linked to increased multiple chronic diseases, comorbidities and mortality (13–15).

Frailty is a clinical condition highly prevalent in the aged population in which a frail individual is more vulnerable to health-related risk exposure (16, 17). Studies showed that this condition has been linked to increased hospitalizations, Emergency Department (ED) visits (18–21), poorer quality of life (22), impaired cognitive function (23), increased morbidity and mortality (24). Frailty is commonly defined by Fried et al. using unintentional weight loss, gait speed, exhaustion, grip strength and physical activity as a clinical diagnostic criteria (25).

There has been an increase in studies on frailty in recent years since frailty could be decreased or reversed with a long-term-based exercise intervention (26, 27). Fairhall et al. conducted an randomized controlled trial of 241 community-dwelling older adults in Australia where the findings showed that exercise and nutrition intervention could significantly improve frailty status in the treatment group (28). The result agreed with Nakamura et al. where 111 community-dwelling older people in Japan were randomly assigned to perform a home-based training during Covid-19 pandemic (29).

Several studies have employed digital tools to help diagnose and treat frailty as technology has improved and become more accessible (30, 31). We believe that information technology can help us recognize frailty earlier, and that the earlier we identify this condition, the better healthcare providers can treat the patient with a better prognosis and health outcomes. This review aims to identify and summarize prospective characteristics, diagnostic models, and therapeutic studies in utilizing digital health technologies in community-dwelling frail older persons.

### Search strategy

We used PubMed as our main source of published literature for our search strategy. The combinations of search terms were (“frailty*” OR “frail” OR “frail elderly”) AND (“digital” OR “machine learning” OR “smartphone*” OR “AI” OR “artificial intelligence” OR “deep learning” OR “device*”) AND (“older adults” OR “elderly” OR “elder” OR “old”). The selected publications in our review were limited to English publications and publications within 2012–2023. Additional literature found in systemic reviews and meta-analysis were manually selected to include in this review. Inclusion Criteria: (1) The study recruited older adults aged at least over 50 years old; (2) The study applied digital health tools to find association, causal relationship or make prediction between frail and non-frail population. Exclusion Criteria: (1) The study was not written in English; the study did not demonstrate a comparison of result between frail and non-frail population; The study did not utilize digital tools.

### Characteristics studies

We found 9 relevant frailty characteristics studies and summarized them into Table 1. Most studies used various types of digital sensors to measure surrogate outcome of frailty and are categorized into (1) Trunk parameter (2) Gait parameter and (3) Non-gait parameters.

**Table 1:** Characteristics studies.

Author (year)	Study design	Population		Frailty criteria	Tool	Parameters	Outcome			Interpretation
		Study base	Participants				Mean (±SD)		*p*-value	
							Non-frail	Frail		
Galán-Mercant et al. (32)	Measurement of 3D acceleration, angular velocity, and trunk displacement in the turn transition of Sit-to-Stand and Stand-to-Sit Transitions	Community-based	*N* = 30 volunteers aged >60 years old.	FFP	iPhone4 attached at the chest	Sit-to-stand RV acceleration mean	4.12 ± 0.96	2.97 ± 1.06	0.005	Accelerometer parameters and angular displacement are significantly different between NF and F groups
						Stand-to-sit RV acceleration mean	4.26 ± 1.05	3.19 ± 0.71	0.005	
						Sit-to-stand Gyroscope Rotation mean	83.83 ± 150.56	24.75 ± 58.16	0.034	
						Stand-to-sit Gyroscope Rotation mean	83.10 ± 142.18	15.49 ± 40.88	0.038	
Millor et al. (33)	Participants were instructed to stand up and sit down from a chair at their preferred speed as many times as possible within 30 s	Community-based	47 community-dwelling adults age >50 years old	FFP	MTx XSENS worn on lumbar spine	30 s Stand-to-sit cycles (*n*)	22 ± 7	6 ± 1	<0.001	Healthy participant performed Sit-to-stand cycle significantly better than frail participants
Toonsizadeh et al. (34)	Participants performed a 50 s trail of elbow flexion in a seated position in a chair wearing a wireless monitor for data collection.	Community-based	*N* = 117 community dwelling volunteers aged >65 years.	FFP	BioSensics LLC on upper arm naer biceps muscle and wrist	Speed of elbow flexion	1,117 ± 247	461 ± 215	0.001	Non-gait related parameters are also associated with frailty status
						Flexibility of elbow flexion	134 ± 22	87 ± 28	<0.001	
						Power of elbow flexion	205.1 ± 116	23.5 ± 15	<0.001	
Parvaneh et al. (35)	Participants were instructed to wear sensors for 48 h to monitor and assess postural transition differences among frailty levels. The first 24 h were used for data analysis.	Community-based	120 community-dwelling volunteers aged >70 years old	FFP	PAMSys with sensors located at the chest	Sit-to-stand (*n*)	85 ± 45	83 ± 40	–	The number of sit-to-walk and total transition cycles derived from chest sensors are correlated with frailty status
						Sit-to-walk (*n*)	23 ± 11	23 ± 9	0.664	
						Stand-to-sit (*n*)	64 ± 37	66 ± 34	0.568	
						Stand-to-walk (*n*)	475 ± 208	332 ± 148	0.011	
						Quick sitting (*n*)	45 ± 16	40 ± 15	0.570	
						Walk-to-stand (*n*)	453 ± 202	314 ± 141	0.363	
						Total transition (*n*)	1,174 ± 468	878 ± 333	0.032	
Castaneda-Gameros et al. (36)	Participants wore the sensors for 7 days. (at least 10 h a day)	Community-based	*N* = 60 community-dwelluing volunteers aged >60 years old	FFP	Actigraph GT2X wore at the hip	Sedentary time	523.7 ± 85.7	576.7 ± 7	0.480	Moderate-to-vigorous Physical Activity was the only parameter that was significantly different between frail and robust groups
						Low-light physical activity	207.4 ± 57.8	161.4 ± 68.7	0.510	
						High-light physical activity	27.1 ± 13.6	18.4 ± 23	0.360	
						Moderate-to-vigorous physical activity	18.4 ± 19.9	3.4 ± 4.5	<0.001	
Zhou et al. (37)	This study aims to examine whether parameters from an instrumented trail-making task (iTMT), gait speed and power could classify frailty stages	Out-patient clinic	61 community-dwelling volunteers aged >60 years	FFP	iTMT and LEGSys worn on both shins	Gait speed	1.06	0.94	0.032	The study showed that parameters were helpful to discriminate frailty status among the out-patient care patients
						iTMT: Velocity	6.31	5.67	0.025	
						Power	90.56	73.70	0.04	
						Exhaustion	8.23	9.41	0.698	
						Variability	20.92	23.05	0.241	
Jansen et al. (38)	Participants were asked to wear the sensors while performing a walk test under two conditions: (1) at self-selected distance of 4.57 m and (2) ask quickly as possible distance of 10 m.	Community-based	*N* = 112 older adults aged 65 years or older	FFP	PAMSys sensor in a shirt & LEGSys at legs and lumbar spine	Percentage of time walking or standing (%)	25 ± 7.10	16.4 ± 7.30	<0.001	The study showed that percentage of time walking/standing, max step in one test bout, and walking speed are significantly different among frailty status
						Max steps in one bout	1,668 ± 1,724	285 ± 387	<0.001	
						Average steps per bout	39 ± 24	27 ± 12	0.250	
						Normal walking speed	1.18 ± 0.15	0.64 ± 0.25	<0.001	
						Fast walking speed	1.47 ± 0.22	1.07 ± 0.12	<0.001	
Apsega et al. (39)	Participants performed TUG test (3 m) while wearing the sensors	Not specified	*N* = 133 community-dwelling adults aged >60 years	FFP	SHIMMER sensors at bilateral thighs, shins, and feet	TUG time	0.67 (1.89–3.78)^a^		<0.001	TUG, Dynamic gait index score, gait speed, and stride time were correlated with frailty status in community-dwelling older persons
						Dynamic gait Index score	0.71 (0.60–0.83)^a^		<0.001	
						Gait speed	0.92 (0.89–0.95)^a^		<0.001	
						Stride time	1.00 (1.003–1.009)^a^		<0.001	
						Swing Phase	1.00 (1.001–1.015)^a^		0.024	
						Stance phase	1.00 (1.004–1.012)^a^		<0.001	
						Double support time (ms)	1.01 (1.01–1.02)^a^		0.002	
						Candence (step/min)	0.83 (0.78–0.89)^a^		<0.000	
Kikuchi et al. (40)	Association of intensity-specific PA and bout-specific sedentary time with frailty status → wear the device for 7 days	Community-based	511 community-dwelling adults aged >65 years	J-CHS	Active style Pro HJA-750C worn at the hip	Short bout of SB	273.1 ± 65.4	231 ± 59	<0.001	Sedentary behavior and physical activity (moderate-to-vigorous) are significantly different between frail and robust patients
						Prolonged bout of SB	167.3 ± 115.3	289.9 ± 157.7	<0.001	
						Light PA	406.2 ± 97.4	298.6 ± 157.9	0.182	
						Moderate-vigorous PA	58.6 ± 40.1	14.9 ± 21.1	<0.001	

F, frailty; FFP, fried frailty phenotype; J-CHS, cardiovascular health study criteria for Japanese older adults; NF, non-frailty; PA, physical activity; RV, resultant vector; SB, sedentary bout; TUG, time-up-and-go.

^a^ Odd Ratio (95% CI) to be frail.

#### Trunk parameter

Most studies' methods involved researchers instructing volunteers to perform physical function tests while wearing a digital sensor that measures characteristics that likely represents frailty. Galan-Mercant et al. studied 30 community-dwelling volunteers over the age of 60 who performed a sit-to-stand, stand-to-sit test while wearing an iPhone4 attached to the chest to assess 3D acceleration, angular velocity, and trunk displacement during the turn transition (32). The findings revealed all factors differed significantly between frail and non-frail subjects.

Parvaneh et al. conducted a study using a wearable necklace-like sensors located at the chest of 120 community-dwelling participants aged over 70 years old to monitor and assess postural transition differences among frailty levels for 24 h, and the results showed that the number of Stand-to-walk and total postural transitions were significantly different between groups (35). Millor et al. asked 47 community-dwelling volunteers over the age of 50 to perform stand-up and sit-down from a chair as many times as they could in 30 s while wearing an inertial orientation tracking sensors on their lumbar spine (33). The study showed that healthy participants outperformed frail people with less sway on the sit-to-stand cycle.

Therefore, the parameters derived from sensors attached to the trunk such as 3D acceleration, velocity and postural sway while doing physical function tests could discriminate frail and robust in the community-dwelling older adults.

#### Gait parameter

Gait assessment was another method used by researchers to analyze diagnostic variables in frail older adults. Zhou et al. investigated whether parameters from an instrumented trail making task (iTMT) and gait sensors worn on both shins to measure gait speed and iTMT derived parameters could distinguish between frail and robust participants (37). The findings revealed that gait speed and iTMT velocity were significant parameters that could help classify frailty status among the outpatient care population. Moreover, Jasen et al. carried out an intervention research which 112 community-dwelling older persons were requested to wear a wearable sensor in a shirt while undertaking a walking test under two conditions: (1) Walk a distance of 4.57 m at your own speed; and (2) Walking a 10-m distance as rapidly as possible (38). The findings correlated with the previous studies, which suggested that the proportion of time spent walking and standing, the maximum steps in one test bout, and walking speed might all be potential predictors of frailty classification (39).

#### Non-gait parameters

To determine frailty status, other variables could be used in addition to those mentioned above. Toosizadeh et al. studied the association between frailty status and non-gait parameters using a wearable gyroscope sensor attached to the upper arm and wrist of 117 community-dwelling adults over 65 years old to measure elbow function while performing a 50-s trail of elbow flexion in a seated position (34). The results revealed that the speed of elbow flexion, flexibility, and power of elbow flexion differed significantly between robust and frail participants. In the study by Castaneda-Gameros et al., moderate-to-vigorous physical activity measured by a sensor that records acceleration and gyroscopic data worn on the hip for 7 days was associated with frailty status in community-dwelling old adults (36). Additionally, Kikuchi et al. found the association of intensity-specific physical activity. The results showed that sedentary behavior and physical activity (moderate-to-vigorous) were significantly different between frail and robust in 511 Japanese community-dwelling participants aged over 65 (40).

As a result of the mentioned studies, there are multiple potential variables that could represent characteristics of frailty. Non-gait parameters appeared to have the highest clinical feasibility if researchers could integrate a model into a smartwatch since a wrist-worn device is simple to use and most older adults are already accustomed to wearing a smartwatch.

## Diagnostic studies

Frailty identification is a clinically relevant topic since it is a condition that may be reversed from frail to robust. Several studies are being conducted to develop tools and diagnostic models for classifying frail and non-frail older adults. According to the authors’ evaluation of the published evidence in this field, there are two types of frailty diagnostic tools that use technology: (1) Clinical Data; and (2) Data derived from wearable devices and biological sensors which are summarized in Table 2.

**Table 2:** Diagnostic studies.

Author (year)	Study design	Population		Predictors	Frailty-criteria	Tool	Model	Diagnosis performances	Interpretation
		Study base	Participants						
Prediction model									
Aznar-Tortonda et al. (41)	Cross-sectional observational study using an application for Android for data collection	Community-based	621 older adults	Sex, age, polypharmacy, hospital admission in the last year, and diabetes.	FFP	Android Application	LGR	AUC 0.78	Simple clinical history could be used for frailty classification in older adults
Sajeev et al. (42)	Cross-sectional Observational study (Development and internal validation with test sample)	Community-based	656 independent community-dwelling adults aged 40–75 years old	63 anthropometric, environmental, social, lifestyle and physiologic variables	CFS and FFP	In-person Health Assessment	LGR	AUC 0.69	Machine learning methods are useful for frailty diagnosis however some variables might be hard to implement in clinical practice
							LDA	AUC 0.69	
							SVM	AUC 0.69	
							RF	AUC 0.71	
Data derived from wearable devices and biological sensors.									
Greene et al. (43)	Development of classifier models to assess frailty status using sensor-derived features of TUG, Five Time Sit to Stand and Balance tests	Community-based	124 community dwelling older adults (mean age 75.9 ± 6.6 years, 91 female). 66 F, 58 NF	Time up and go test, Balance test, Five Time Sit to Stand	FFP	SHIMMER sensor worn on each shin, right thigh, L5 spine and sternum. A pressure sensor for balance data.	SVM	Sensitivity 88.63% Specificity 85.06%	TUG, FTSS and Balance test are good predictors for frailty classification using an SVM model
Schwenk et al. (44)	Participant walked 4.57 m in their home at self-selected speed. Balance was assessed during 15 s quiet standing with feet together, eyes closed. PA	Community-based	*N* = 125 community-dwelling volunteers aged >65 years old	Stride length, Double support, Balance parameters	FFP	LEGSys, BalanSens, PAMSys with sensors located at shanks, thighs, and lumbar spine	LGR: NF and PF classification	AUC 0.86	Gait parameters had the best performance to separate NF from PF and PF from F in aged-adjusted model
							LGR: PF and F classification	AUC 0.84	
Tooiszadeh et al. (45)	Participants performed two 15 s balance mechanisms between NF, PF and F individuals	Community-based	122 older adults aged >65 years old	Postural sway, age, BMI, OLCL parameters	FFP	BalanSens at lumbar spine and shin	LGR: Postural sway, age, and BMI	EO: Sensitivity 74%, Specificity 93% EC: Sensitivity 74%, Specificity 83%	Body sway (and age/BMI), OLCL (and age/BMI) can be used for frailty screening tool (high sensitivity)
							LGR: OLCL, age and BMI	EO: Sensitivity 94%, Specificity 98% EC: Sensitivity 100%, Specificity 83%	
Tooiszadeh et al. (46)	Validate the accuracy of Upper-Extremity-Frailty (UEF) assessment in distinguishing between F and NF participants	Hospital-based	101 hospital in-patients aged >65 years old	Speed of elbow flexion, Number of flexions, Power, and Moment	TSFI	BioSensics LLC: near biceps and wrist	LGR	Sensitivity 78% Specificity 82%	This study shows that a single sensor worn at wrist could be a viable tool for frailty assessment tool however a higher sensitivity would be better on frailty screening use-case.
Millor et al. (47)	Participants performed as many CST reps as possible within 30 s at self-selected speed starting from seated position with arms folded	Not specified	A total of 718 subjects from an elderly population aged over 70 years	Temporal-spatial gait parameters: Gait Velocity, Step Regularity, Stride Regularity, Symmetry, Step Time variability	FFP	MTx XSENS worn on L3 spine	Decision Tree: GV	AUC 0.82	The results showed that the sensors are useful for frailty classification using gait parameters
Lee et al. (48)	Participants wore sensors while performing elbow flexion and extension in 20 s timeframe to provide physical frailty assessment features	Hospital-based	*N* = 100 in-patients (old adults) aged over 70 years old	Mean of angle range, PD of power range, CV of elbow extension time, mean of elbow flexion time, CV of elbow flexion time	Rockwood's criteria (TSFI)	LEGSys worn at wrist and upper arm	LR	AUC 0.87	This study shows that a single sensor worn at wrist could be a viable tool for frailty assessment tool

AUC, area under the receiver operating characteristic curve; BMI, body mass index; CFS, clinical frailty scale; CST, chair-sit-test; CV, coefficient of variation; EC, eyes closed; EO, eyes open; F, frailty; FFP, fried frailty phenotype; FTSS, five time sit to stand; GV, gait velocity; LDA, linear discriminant analysis; LGR, logistic regression; LR, linear regression; NF, non-frailty; OLCL, open-loop close-loop; PD, percentage of decline; PF, pre-frail; RF, random forest; SVM, support vector machine; TSFI, rockwood's criteria; TUG, time-up-and-go.

### Clinical data

Aznar-Tortonda et al. collected data from 621 community-based participants in a cross-sectional observational study utilizing an Android mobile device application. Sex, age, polypharmacy, hospitalization, and diabetes history were chosen characteristics and employed in a logistic regression model (41). This model obtained an AUC of 0.78, suggesting that a brief clinical history might be utilized to classify frailty in older persons. Sajeev et al. used 20 anthropometric, environmental, social, lifestyle, and physiologic variables from 656 community-dwelling adults aged 40–65 years old to develop and internally validate four machine learning models, including logistic regression, linear discriminant analysis, support vector machine, and random forest (42). With an AUC of 70.8, the random forest model achieved the highest discrimination performance. This study found that machine learning models could be used to diagnose frailty. However, the large number of variables in the purposed models could make it difficult to implement them in clinical practices and community settings, and the selected features appeared to be more difficult to measure and more complicated than the standard diagnostic criteria for frailty. A future study is required to demonstrate the real-world application of a frailty diagnostic machine learning model based on clinical characteristic data.

#### Data derived from wearable devices and biological sensors

Most studies for biological sensors and wearable devices employ criteria similar to characteristics research. We divided the parameters into three major categories: (1) Physical Function test; (2) Gait and balance test; and (3) Non-gait-related test.

##### Physical function test

Greene et al. created a support vector machine classifier model based on characteristics gathered from 124 community-dwelling people who wore inertial and pressure sensors on each shin, right thigh, L5 spine, and sternum while undertaking Time-up-and-go, Five Time Sit to Stand, and Balance tests (43). Their model had 88.63% sensitivity and 85.06% specificity, indicating that the demonstrated tests had good frailty classifying characteristics. Schwenk et al. had 125 community-dwelling older adults walk 4.57 m in their home at their own pace, followed by a balance assessment while wearing multiple sensors on their shanks, thighs, and lumbar spine to collect gait and balance parameters for logistic regression model development (44). The results revealed an AUC of 0.857 for non-frail and pre-frail classification and an AUC of 0.841 for pre-frail and frail classification. The mentioned models have shown good and applicable discrimination performance.

##### Gait and balance test

Tooiszadeh et al. demonstrated that postural sway, age, and BMI parameters derived from sensors located at the lumbar spine and shin could predict frailty with 97% sensitivity and 88% specificity (45). Millor et al. developed decision tree models using gait characteristics acquired from an inertia sensor worn on the L3 spine of 718 senior volunteers aged over 70 years (47). With an AUC of 0.823–0.896, the results also demonstrated that gait characteristics and decision tree models were beneficial for frailty classification.

##### Upper extremity

According to Lee et al., participants wore accelerometers and gyroscope sensors at their wrist and upper arm while performing elbow flexion and extension in a 20-s timeframe to provide physical features such as the mean of the angle range coefficient of variation of elbow flexion and extension time and the mean of elbow movement time (48). These characteristics were used to develop a linear regression model with an AUC of 0.87. Tooiszadeh et al. created a logistic regression model utilizing upper-extremity frailty assessment data from a wearable gyroscope sensor, which was collected from the upper extremities of 101 hospital in-patients over the age of 65 (46). The study's performance was 78% sensitivity and 82% specificity. These studies demonstrated that a single non-gait-related sensor could be used to distinguish frailty and robustness in the elderly population.

In conclusion, research revealed that physical function tests, gait-related, and non-gait-related measures were useful in developing prediction models to diagnose frailty state in the aged population. However, the fitness test approach may be unsuitable for prospective frailty data collection because performing all the aforementioned fitness tests would take a significant amount of time to obtain the required feature in order to diagnose frailty in an individual, which may be comparable to simply performing tests according to Fried's criteria. We propose that future research should focus on upper extremity features because we believe that integrating a frailty predictive model into a smartwatch and mobile application has clinically significant implications.

## Therapeutic studies

Based on the current evidence summarized in Table 3, pre-frail and frail older adults are recommended for multi-component physical activity program and progressive resistance training program. Multiple studies have shown improved cognitive function, physical function, and frailty status in older adults after physical exercise intervention. Therefore, our review selected frailty therapeutic studies that integrated the use of technology to improve frailty state in the elderly.

**Table 3:** Therapeutic studies.

Author (year)	Study design	Participants	Control group	Intervention group	Frailty criteria	Tool	Qualitative outcome	Quantitative outcome
Daniel et al. (49)	Pre-frail volunteers were recruited to participate in a 15-week exercise intervention or control group. Participants were randomized into one of three groups: control, seated exercise, or Wii®-fit.	23 Community-based pre-frail participants aged over 70 years old	Two intervention groups: (1) Wii®-fit. exercise at home and (2) seated exercise (with trainers)	Normal physical activity	FFP	Wii®-fit	Better outcomes for the intervention group. Wii-fit exercises and seated exercises were both superior to the control group in maintaining or improving physical functioning.	Time up and go test remain the same in control group while the treatment group had increased ES = 0.27 (Seated exercise) and 0.30 (Wii)
Takahashi et al. (50)	Participants were randomized to telemonitoring (with daily input) or to patient-driven usual care. Telemonitoring was accomplished by daily biometrics, symptom reporting, and videoconference. The primary outcome was a composite end point of hospitalizations and ED visits in the 12 months following enrollment.	102 frail individuals with multiple comorbidities	Telemonitoring	Usual care	ERA	Intel® health guide and other medical equipment at home	No difference between groups in most of the outcome measurements	ES for main outcome = 0.0991
Upatising et al. (51)	–	194 participants aged over 70 years old with different frailty status and chronic conditions	The intervention group received usual medical care and telemonitoring case management	Usual care	FFP	Intel® health guide and other medical equipment at home	No difference between group	No transition to a frailty state during the first and the subsequent 6 months (OR 1.41, 95% CI 0.65–3.06, 5.94, 95% CI 0.52–68.48)
Dekker-van Weering et al. (52)	Participants were randomly assigned to a control group or a 12-week intervention group. Primary outcomes were use of the intervention, adherence to a 3-day exercise protocol and user experience [System Usability Scale (SUS); rating 1–10].	36 prefrail individuals with mean age 70.9	Home exercise program using computer/tablet, 3 times a week for 12 weeks	Usual care	GFI	Home exercise program (strength, balance, and flexibility exercises)	The study showed that the programs are feasible and easy to use for pre-frail elderly adults	Acceptability: average score SUS 84.2 (±13.3). Adherence: 68%. Quality of life (mental) better in intervention group, other quality of life domains, no difference.

CI, confidence interval; ED, emergency department; ERA, elder risk assessment index; ES, effect size; FFP, fried frailty phenotype; GFI, groninger frailty indicator; OR, odd ratio.

Daniel et al. conducted a study where 23 community-dwelling pre-frail volunteers aged over 70 years old were randomized into one of three groups: control, seated exercise, or Wii®-fit. The findings showed better outcomes for all intervention groups (49). Wii-fit exercises and seated exercises were both superior to the control group in maintaining or improving physical functions. Liao et al. recruited a randomized controlled trial of 52 prefrail and frail elderly where the participants were divided into two exercise intervention (1) Exergaming group and (2) Combined resistance, aerobic and balance exercise group for 36 sessions over 12 weeks (53). The results revealed both gaming exercise and combined exercise groups improved frailty status among the elderly. The study correlated with Moreira et al. where an RCT of 66 pre-frail older adults were assigned to either exergaming intervention and traditional multicomponent exercise (54). The findings showed that both programs were clinically effective for delaying frailty status and improving physical and cognitive function.

Exergaming have shown positive health outcomes in terms of enhancing physical function, cognitive function, and frailty status. The programs could be done in a home setting, making exercise intervention easily accessible. However, the majority of frail people are older adults, who may face challenges using technologies because of their lack of digital literacy and technology acceptance. One of the studies cited above had a dropout rate of over 30%, which suggests that a portion of older persons might not find the use of a digital intervention tool appropriate.

## Discussion

From our review, we found that there are many potential etiognostic factors that could help diagnose frailty status using digital tools from trunk, gait, upper-extremity, and physical activity parameters. Researchers had used these parameters to create multiple well-performing models to classify frailty status in the older adults. We found non-gait parameters the most appealing variables for future research as a frailty diagnostic model integration into a wearable device. However, the model classification results should be interpreted with caution because these models may be overfitting due to a lack of external validation studies.

Regardless of the tools used, studies have shown that exercise can improve frailty status. Rather than developing a single standalone exercise platform, digital health technology developers should focus on how to implement these therapeutic platforms with health care providers or coaching platforms that could encourage and motivate prefrail and frail old adults to engage in more physical activity.

Integrating digital health tools into frailty diagnosis and management presents challenges, particularly in terms of adoption among older individuals. A study showed that Frailty was linked to both physical activity and technology adoption (55). In order to counteract frailty, this study suggests that older persons who are less receptive to technology engage in physical exercise. Another study showed that, whereas elderly people use mobile phones extensively, wearable device adoption is low and 63.2% of surveyed participants were unable to install or delete applications independently. Furthermore, pre-frail and frail older persons use healthcare apps more frequently than their healthy colleagues, showing a significant desire for health-related services situation, helping individuals enhance their health and cognitive abilities (56). This encourages researchers to develop solutions using digital health tools for frail older adults. However, the solutions should also be both user-friendly, gamified and engaging, ensuring active involvement and adherence for older populations.

Developing comprehensive platforms that integrate screening with therapeutic recommendations, such as apps providing tailored guidance on physical activity, diet, and medical consultations based on clinical guidelines for frailty might serve as a single resource for early screening and frailty intervention (57, 58). This could fit in the healthcare system by enabling early detection and giving interventions to the individual with risks, reducing the burden on physicians and patients by stratifying risks for efficient healthcare human resources management, and can be integrated with hospital systems to streamline care using data-driven insights from the frailty risk assessment models.

For instance, a study that developed and validated a fitness application for specific populations, like seafarers, demonstrated that they can improve physical activity and health outcomes by providing tailored physical training programs suitable for the maritime environment (59). This bridge the gaps between technology, frail individuals, healthcare professionals, and caregivers.

## Conclusion

In conclusion, the review highlights the promising role of digital health tools in addressing frailty among older adults. The use of sensor-derived metrics for upper extremity, trunk, and gait evaluation has improved the early detection of frailty and provided useful intervention options. Furthermore, therapeutic applications, such as exergaming and home-based programs, have demonstrated significant improvements in physical and cognitive functions, albeit with challenges related to technology acceptance among older adults. Our study underlines the necessity for future research to bridge the gap between frailty screening and therapeutic interventions by developing comprehensive, user-friendly digital platforms that combine diagnosis with personalized preventive care. This integrated approach has the potential to enhance health outcomes and quality of life for the aging population.
